# Optical sensing of anticoagulation status: Towards point-of-care coagulation testing

**DOI:** 10.1371/journal.pone.0182491

**Published:** 2017-08-03

**Authors:** Diane M. Tshikudi, Markandey M. Tripathi, Zeinab Hajjarian, Elizabeth M. Van Cott, Seemantini K. Nadkarni

**Affiliations:** 1 Wellman Center for Photomedicine, Massachusetts General Hospital, Harvard Medical School, Boston, MA, United States of America; 2 Department of Pathology, Massachusetts General Hospital, Harvard Medical School, Boston, MA, United States of America; Pennsylvania State Hershey College of Medicine, UNITED STATES

## Abstract

Anticoagulant overdose is associated with major bleeding complications. Rapid coagulation sensing may ensure safe and accurate anticoagulant dosing and reduce bleeding risk. Here, we report the novel use of Laser Speckle Rheology (LSR) for measuring anticoagulation and haemodilution status in whole blood. In the LSR approach, blood from 12 patients and 4 swine was placed in disposable cartridges and time-varying intensity fluctuations of laser speckle patterns were measured to quantify the viscoelastic modulus during clotting. Coagulation parameters, mainly clotting time, clot progression rate (α-angle) and maximum clot stiffness (MA) were derived from the clot viscoelasticity trace and compared with standard Thromboelastography (TEG). To demonstrate the capability for anticoagulation sensing in patients, blood samples from 12 patients treated with warfarin anticoagulant were analyzed. LSR clotting time correlated with prothrombin and activated partial thromboplastin time (r = 0.57–0.77, p<0.04) and all LSR parameters demonstrated good correlation with TEG (r = 0.61–0.87, p<0.04). To further evaluate the dose-dependent sensitivity of LSR parameters, swine blood was spiked with varying concentrations of heparin, argatroban and rivaroxaban or serially diluted with saline. We observed that anticoagulant treatments prolonged LSR clotting time in a dose-dependent manner that correlated closely with TEG (r = 0.99, p<0.01). LSR angle was unaltered by anticoagulation whereas TEG angle presented dose-dependent diminution likely linked to the mechanical manipulation of the clot. In both LSR and TEG, MA was largely unaffected by anticoagulation, and LSR presented a higher sensitivity to increased haemodilution in comparison to TEG (p<0.01). Our results establish that LSR rapidly and accurately measures the response of various anticoagulants, opening the opportunity for routine anticoagulation monitoring at the point-of-care or for patient self-testing.

## Introduction

Thrombotic and thromboembolic events are the most common causes of morbidity and mortality [1]. In many cases, thromboembolic disorders may be preventable and treatable with effective anticoagulant therapy [2,3]. Parenteral and oral anticoagulants such as heparin, argatroban, warfarin, rivaroxaban, and several other drugs are widely prescribed during peri-operative procedures and for acute or long-term treatment in tens of millions of patients worldwide [2]. Despite their effectiveness in preventing and treating thromboembolic events, anticoagulant therapies, even when maintained within therapeutic ranges, are often associated with major bleeding complications including haemorrhagic shock [1,4]. Acute bleeding events may require fluid resuscitation leading to extensive haemodilution, complex coagulopathy and significant morbidity and mortality [4–6].

Anticoagulation-associated bleeding can arise due to long-term use, overdose, urgent surgeries, during transition between anticoagulants and in cases of recurrent thrombosis [4,7]. Anticoagulation management in these patients is challenging because a narrow therapeutic window often exists between bleeding and coagulation. Anticoagulation is further influenced by numerous food and drug interactions, hepatic or renal impairment and the variability in dose response [4,8,9]. As a result, clinicians routinely walk a thin line to maintain a delicate balance between bleeding and thrombosis. Consequently, most patients require frequent laboratory testing of blood coagulation status to ensure accurate and safe anticoagulant dosing [3,7]. Traditionally, laboratory-based coagulation assays such as activated partial thromboplastin time (aPTT), prothrombin time (PT), activated clotting time (ACT) and chromogenic anti-Xa assays are commonly used to monitor anticoagulants therapies [10]. Laboratory testing however has long turn-around times and can be expensive over time, placing a large burden on health care resources [1,8]. Recently, to meet the need for comprehensive point-of-care (PoC) testing, viscoelastic assays such as rotational thromboelastometry (ROTEM) and thromboelastography (TEG) have provided rapid alternatives to routine laboratory testing by allowing assessment of global haemostasis in real-time [11,12]. Yet, several concerns including the need for daily calibration and specialised operators, mechanical manipulation of the clot, the long data reporting time, large instrument size, high cost and the lack of standardised procedures have limited the widespread adoption of TEG and ROTEM for routine anticoagulation assessment at the PoC [13].

We have recently developed a new optical sensor that utilizes Laser Speckle Rheology (LSR) approaches to rapidly quantify a patient’s coagulation status using a few drops of whole blood by measuring changes in blood viscoelasticity during coagulation from a time series of laser speckle patterns [14–17]. Laser speckle that occurs by the interference of scattered laser light, is exquisitely sensitive to the Brownian motion of endogenous light scattering particles in turn influenced by the viscoelastic susceptibility of the medium [14–18]. The increasing stiffness of blood during coagulation therefore elicits a slower rate of speckle fluctuations in a clot compared with un-clotted blood [14,15]. In a recent study we have shown that clotting time and clot stiffness measured by LSR are closely correlated with plasma-based laboratory tests of aPTT, PT and fibrinogen levels in patients with a range of coagulation abnormalities [14]. The goal of the current study is to investigate the capability of using LSR as a tool to quantify anticoagulation status in response to treatment via four common classes of anticoagulants. We first conducted a pilot clinical study to demonstrate the capability for monitoring anticoagulation in patients treated with warfarin anticoagulant, a common Vitamin K antagonist (VKA). Next, using swine blood, we assess the accuracy and sensitivity of LSR in measuring the dose-dependent response of several common anticoagulants including an indirect thrombin inhibitor (heparin), a factor Xa inhibitor (rivaroxaban) and a direct thrombin inhibitor (argatroban) via comparison with standard reference TEG measurements. Finally we also evaluate the sensitivity and accuracy of LSR to identify coagulation changes due to haemodilution often associated with fluid resuscitation in patients.

## Materials and methods

### Blood sample collection and preparation

Patient blood samples: The use of patient blood samples was approved by the Institutional Review Board of the Massachusetts General Hospital. De-identified whole blood samples from 12 patients receiving oral warfarin therapy (Coumadin) and undergoing conventional coagulation testing were collected in 0.105M sodium citrate Vacutainer system from the MGH special coagulation laboratory. In these patients laboratory tests of aPTT, PT, and INR were conducted as per clinical standard-of-care.

Swine blood samples: The study was approved by the Institutional Animal Care and Use Committee of Massachusetts General Hospital (MGH). Fresh blood samples from 4 female Yorkshire swine were drawn from a central venous catheter line using a Vacutainer system containing 0.105M sodium citrate (Becton Dickinson, Franklin Lakes, NJ, USA). Due to the short stability period of whole blood (~7 hours) for coagulation assessment and the long measurement time required for each TEG analysis (up to 60 min), it was difficult to evaluate more than one anticoagulant or hemodilution treatment from the same blood draw. Therefore, in this study, each of the 4 treatments (heparin, rivaroxaban, argatroban or hemodilution) with the corresponding dose response was conducted per swine, which required 4 swine for use in the study. To evaluate dose-dependent anticoagulation using LSR, swine whole blood samples were spiked with 5 μL of heparin (0.1, 0.2, 0.25, 0.3 USP/ml) [19,20], argatroban (3.8, 5.7, 7.6, 15.2 μM) [21] or rivaroxaban (0.46, 1.15, 1.73 and 2.29 μM) prepared as described below. The anticoagulant doses investigated in the current study were selected based on clinical target ranges generally recommended for prophylaxis for preventing thromboembolic events in patients [7,21–24]. Life-threatening bleeding events may require fluid resuscitation that can further result in extensive hemodilution, leading to complex coagulopathy and significant morbidity and mortality [6]. Therefore, we further assessed the influence of haemodilution on LSR results. To this end, citrated swine whole blood samples were serially diluted at varying concentrations (0–70%) of 0.9% saline solution (Hospira. Inc., Lake Forrest, IL, USA).

Both LSR and TEG testing was performed on all the patient and swine whole blood samples. In all cases whole blood samples were citrated, maintained at room temperature (25°C) and evaluated within less than 4 hours [25].

### Anticoagulants

To test the dose-dependent anticoagulation response using LSR in swine blood, anticoagulant agents were prepared as follows. One rivaroxaban (a common factor X inhibitor) pill of 10 mg (Xarelto ^(R)^, Bayer HealthCare AG, Leverkusen, Germany) was ground to a fine powder and mixed with distilled water to obtain a final concentration of 100 μg/ml. The stock rivaroxaban solution was ultrasonicated for 10 minutes in a water bath at room temperature and was further incubated in a 50°C water bath for 3 minutes [21]. Argatroban (a common direct thrombin inhibitor) solution of 1898.8 μM (The Pharmaceuticals, Inc., Woodcliff Lake, NJ, USA) and heparin at 1000 USP/ml (unfractionated porcine heparin, Sagent Pharmaceuticals, Schaumburg, IL, USA) were also used in this study.

### TEG and LSR coagulation assays

In this study, TEG was used as the standard-reference technique for comparison with LSR because it quantifies coagulation parameters based on a clot viscoelasticity profile similar to LSR. TEG measurements were performed following the manufacturer’s instructions (Haemonetics Inc., Braintree, MA, USA). Briefly, 1 mL of citrated whole blood (non-treated or treated) was mixed with kaolin to activate the intrinsic or contact coagulation pathway (Haemoscope Corporation, Chicago, IL, USA) and 340 μL of the activated blood sample loaded into a TEG measurement cup containing 20 μL of 0.2M calcium chloride (CaCl_2_). The coagulation process was recorded by the TEG analyzer (TEG 5000; Haemoscope Corporation, Chicago, IL, USA) for 30–60 minutes. Subsequently, the remainder of the kaolin-activated blood (660 μl) was recalcified with 38.8 μl of 0.2M CaCl_2,_ and 127 μl of recalcified blood was placed in a disposable imaging cartridge (Grace Biolabs, Bend, OR, USA) (dimensions: 9 mm diameter, 2 mm depth) for LSR analysis (detailed below). All experiments were conducted in triplicate using both LSR and TEG.

### LSR instrument

**Fig 1:**
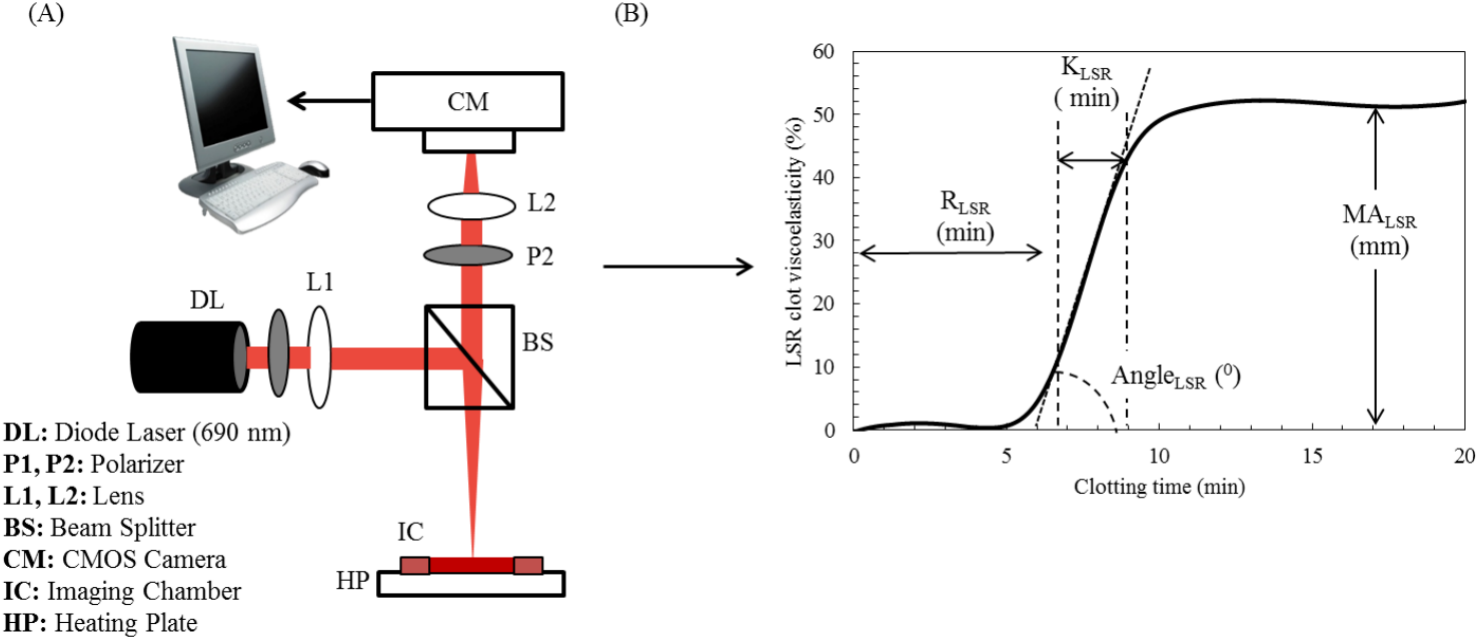
Laser speckle rheology (LSR) instrument and coagulation parameters. (A) The schematic diagram of the LSR instrument used for blood coagulation assessment. Polarized light (690 nm, 9 mW) from a diode laser (Newport Corp., LPM690-30C) was focused (spot size 100 μm) on the imaging chamber containing 127 μl of kaolin-activated blood. Cross-polarized laser speckle patterns were acquired at 180° back-scattering geometry via a beam-splitter using a high speed CMOS camera (Basler AG, acA2000-340km) equipped with a focusing lens (Edmund Optics, NT59-872)[14]. The captured speckle patterns were transferred to a computer for further processing. (B) Representative clot viscoelasticity profile derived using LSR. The relative change in clot viscoelasticity (*G*) is measured during coagulation and plotted as a function of time to retrieve the LSR coagulation parameters, R, K, α-angle and MA. Reprinted from [Tripathi MM, Hajjarian Z, Van Cott ME, and Nadkarni KS. Assessing blood coagulation status with laser speckle rheology. Biomed. Opt. Express. 2014. 5: 817–831] under a CC BY license, with permission from [The Optical Society], original copyright [2014].

LSR measurements were performed using an optical set up detailed in our previous studies (Fig 1A) [14,15]. The recalcified sample loaded in the disposable cartridge was maintained at 37°C with a customized heat plate. The sample was illuminated with a diode laser (690 nm, 11 mW) and focused to a 100 μm (diameter) spot on the transparent optical window of the loaded blood cartridge. The diameter of the laser spot was calculated using the beam propagation of the Gaussian beam model when the laser wavelength is 690 nm, the focal length of the focusing lens is 23 cm and the beam size diameter from the laser output is 2 mm.

A time-series of cross-polarized, laser speckle patterns reflected from the blood sample were acquired using a high speed CMOS camera (Ace 2000–340 km, Basler, Ahrensburg, Germany) and pattern acquisition was conducted at a frame rate of 753 frames/s for 1s at a time, with a 30s time lapse over a duration of 20 minutes. However, due to significant multiple scattering within the illuminated sample, the scattered and returning light interferes at the surface, forming a laser speckle pattern diameter that covered a field of ~8mm. Therefore, the imaging region of interest (ROI) was 500 X 500 pixels covering an area of 8 X 8 mm on the sample. The captured speckle patterns were transmitted to a desktop computer and processed to retrieve the LSR clot viscoelasticity profile and quantify coagulation parameters as summarized below (Fig 1B).

### LSR data analysis

To retrieve the clot viscoelasticity profile, the complex viscoelastic modulus, G*(ω), of blood as a function of frequency, *ω*, was first quantified from time-varying laser speckle intensity fluctuations using algorithms that have been previously described in detail [15,17,18]. Briefly, to calculate G*(ω), of blood, the speckle intensity autocorrelation curve, g_2_(t), was first calculated by performing a 2-dimensional cross-correlation analysis between first speckle frames with subsequent frames of the speckle image time series as [15]

$$g_{2}(t)={\left\langle \frac{{\left\langle {I(t}_{0}{)I(t}_{0}+t)\right\rangle }_{\mathrm{pixels}}}{\sqrt{{\left\langle {I(t}_{0}{)}^{2}\right\rangle }_{\mathrm{pixels}}{\left\langle {I(t}_{0}+{t)}^{2}\right\rangle }_{\mathrm{pixels}}}}\right\rangle }_{t_{0}}$$ (1)

Here *I(t*_*0*_*)* and *I(t*_*0*_*+t)* defines the speckle intensities at times *t*_*0*_ and *t*_*0*_*+t*, and < >_pixels_ and < >_*t0*_ indicates spatial and temporal averaging over all the pixels (500 x 500) and for the duration of speckle time series (1s) respectively. Next, the extent of Brownian displacements of light scattering particles (RBC’s, platelets etc.) defined by the mean square displacement (MSD) was calculated from the measured g_2_(t) as follows[26,27]:

$$g_{2}(t)=e^{\text{-}2\gamma \sqrt{k^{2}\langle \Delta r^{2}(t)\rangle }}$$ (2)

where <*Δr*^*2*^*(t)>* represents MSD, k is the wave vector in the scattering medium which can be further expressed as *k = 2πn/λ*, *n* (= 1.36) is the refractive index of the blood, *λ* (= 690nm) is the wavelength of the incident laser light and *γ* (= 5/3) is an experimental parameter related to the source-detector distance and polarization state of light [15,18,28,29].

As previously described, the MSD calculated above quantifies the random Brownian diffusion of scattering particles in response to thermal forces and is therefore directly linked to the viscoelastic modulus of blood, denoted by G*(ω). The MSD values measured at short, intermediate and long durations correspond to the high, intermediate and low frequency response of the viscoelastic material [26]. It has been previously established that the viscoelastic modulus and the MSD of particles undergoing Brownian motion are related through the Generalized Stokes-Einstein Relation (GSER) as follows [15]:

$$G^{*}\left(\omega \right)=\frac{K_{b}T}{a\pi \left\langle {\Delta r}^{2}(1/\omega )\right\rangle \Gamma \left[1+\alpha (\omega )\right]}$$ (3)

where *K*_*b*_ is the Boltzmann constant (= 1.38 x 10^−23^ m^2^ kg s^-2^ K^-1^), and T is the temperature in Kelvin (= 310°K), *ω = 2πυ = 1/t* represents the angular frequency, *υ* represents frequency, t is time in sec, *Γ* denotes the gamma function and *α*(*ω*) = ${\left|\frac{dln\left\langle \Delta r^{2}(t)\right\rangle }{d\ln t}\right|}_{t=1/\omega }$ denotes the MSD slope in a log-log plot. To compute the absolute value of the viscoelastic modulus, G*(ω), via the GSER, knowledge of the particle radius, *a*, of light scattering particles is required. During coagulation, however, the effective radius of light scatterers is consistently altered with the formation of fibrin monomers and due to platelet aggregation. As a result, an accurate estimate of ‘*a*’ is difficult to obtain. Instead, we measured the quantity *G* at a frequency of *ω* = 5Hz to indicate clot viscoelasticity, where G = a×|G*(ω)|, was equal to the product of the viscoelastic modulus and the particle radius, *a*. Using this approach, we have previously established that LSR can accurately quantify the time course evolution of the viscoelastic modulus during the process of blood coagulation [15].

Next, the time course of the modulus, *G*, was plotted as a function of coagulation time, *t*, and normalized to the baseline value to obtain the LSR amplitude curve, from which the following coagulation parameters were extracted: reaction time (R), kinetic time (K), clotting time (R+K), angle (α), and maximum clot stiffness or maximum amplitude (MA) (Fig 1B and Table 1). The R-time was defined as the time at which the tangent drawn to the rising slope of the LSR amplitude curve intersected with the time axis. The K-time was time between the R-time and the time at which slope of the LSR amplitude curve attained a maximum value. The α-angle was defined as the angle between the tangent and the time axis and the MA was equal to the maximum value of the LSR amplitude curve. The maximum amplitude (MA) represents a measure of clot viscoelastic modulus or clot strength and is related to the interaction between the fibrin network and the activated platelets and fibrin polymerization as discussed further below [30]. All coagulation parameters measured by LSR were then compared with standard TEG results.

**Table 1:** LSR coagulation parameters: Descriptions and definition.

LSR parameters	Definitions
Reaction time (R)	Activation of clotting proteins and initial fibrin formation.
Kinetic time (K)	Platelet activation and amplification of thrombin formation.
Clotting time (R+K)	Time to maximum fibrin formation.
Angle (α)	Clot progression rate.
Maximum Amplitude (MA)	Maximum clot stiffness.

### Statistical analysis

Linear regression analysis using the parametric Pearson correlation coefficient was used to evaluate the correlation between LSR and TEG coagulation parameters, and between LSR clotting time and aPTT and PT. The strength of the correlation for absolute values of r between 0.40 and 0.59 was considerated ‘moderate’ whereas r between 0.6 and 0.79 was defined as having a ‘strong’ or ‘good’ correlation [31]. One-way and two-way analysis of variance (ANOVA) followed by the Tukey’s method for multiple test comparisons (Prism software, GraphPad, San Diego, CA, USA) were performed to measure sensitivity to dose-dependent anticoagulation. In all cases P<0.05 was considered statistically significant.

## Results

### Clinical testing using LSR in warfarin-treated patients: Comparison with TEG and conventional coagulation tests

**Fig 2:**
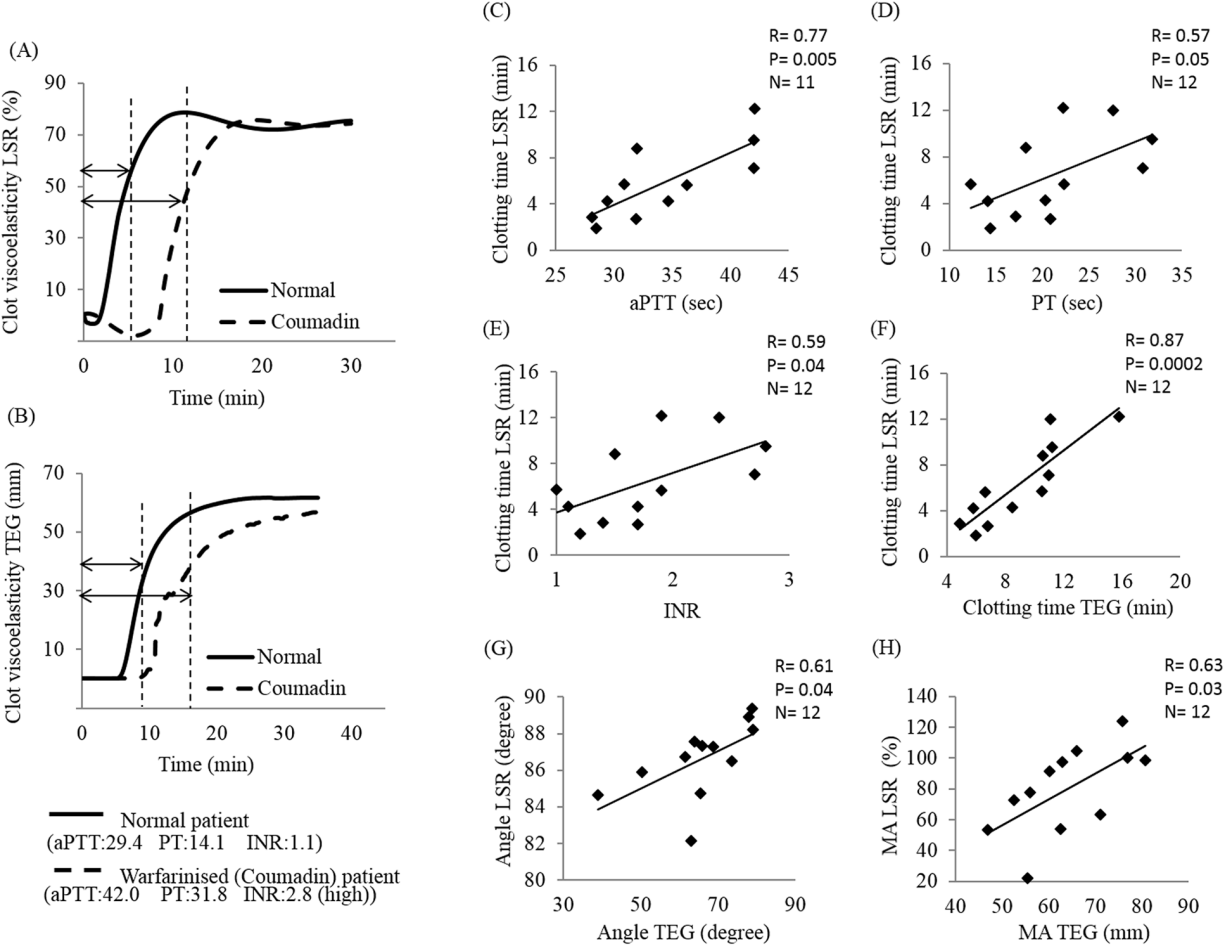
Effect of warfarin treatment on correlations between LSR and aPTT, PT, INR and TEG parameters. Coagulation profiles of 12 patients on warfarin therapy were evaluated using LSR and TEG, and with aPTT and PT/INR assays. LSR clotting time (R+K) parameter was compared to aPTT (A), PT (B), INR (C), and TEG R+K time (D). Furthermore LSR and TEG parameters angle (E) and MA (F) were compared. In Figs (B-F) data from all 12 patients is reported. For Fig (A), N = 11 patients are reported as aPTT was not obtained for one patient.

Fig 2 shows the results of the clinical study conducted to test the capability of LSR in detecting anticoagulation effects of warfarin in patients undergoing conventional coagulation testing (CCT) per standard-of-care. As observed in Fig 2A and 2B, treatment with warfarin affected the LSR coagulation profile similar to TEG via an increase in the clotting time reported for the patient with abnormally high CCT clotting times compared with the normal patient. The normal ranges for CCT clotting times are: aPTT: 22–35 second, PT: 11.5–14.5 second and INR: 0.9–1.1. The pooled data for all patients is shown in Fig 2C–2H. A high correlation was observed between LSR clotting time and aPTT (r = 0.77 p<0.01), which, similar to LSR (and TEG), relies on a kaolin based buffer to activate coagulation via the intrinsic coagulation pathway (Fig 2C). Furthermore, the correlation between LSR and PT was r = 0.57 (p = 0.05) and with INR was r = 0.59 (p = 0.04) (Fig 2D and 2E). Since the PT/INR assay utilizes tissue factor to activate the extrinsic coagulation pathway as opposed to the intrinsic pathway coagulation (measured by aPTT and LSR), only a moderate correlation with LSR was observed. In comparison with TEG coagulation parameters measured from patient samples (Fig 2F–2H), LSR clotting time presented an excellent correlation with the corresponding TEG clotting time (r = 0.87, p<0.001) (Fig 2F). Moreover, a good correlation was observed between LSR and TEG measurements of α–angle (r = 0.61 p = 0.04) (Fig 2G) and MA (r = 0.63 p = 0.03) (Fig 2H). These results demonstrate the capability of LSR in quantifying anticoagulation response in patients similar to standard TEG and CCTs. For the pilot clinical study in patients, the sample size was confirmed by conducting a power analysis. The sample size calculation showed that 12 participants were sufficient to obtain a statistical power of 80% with a β-risk of 20%, confidence level of 95% and a precision (α-risk) of 5% to detect a correlation of r = 0.57 (which corresponded to the lowest detectable correlation between LSR clotting time and lab values of PT). Therefore, a cohort of 12 participants in this study was sufficient to detect a statistically significant correlation for all parameters as observed in the paper.

### Swine testing: Assessment of dose-dependent anticoagulation using LSR and comparison with TEG

**Fig 3:**
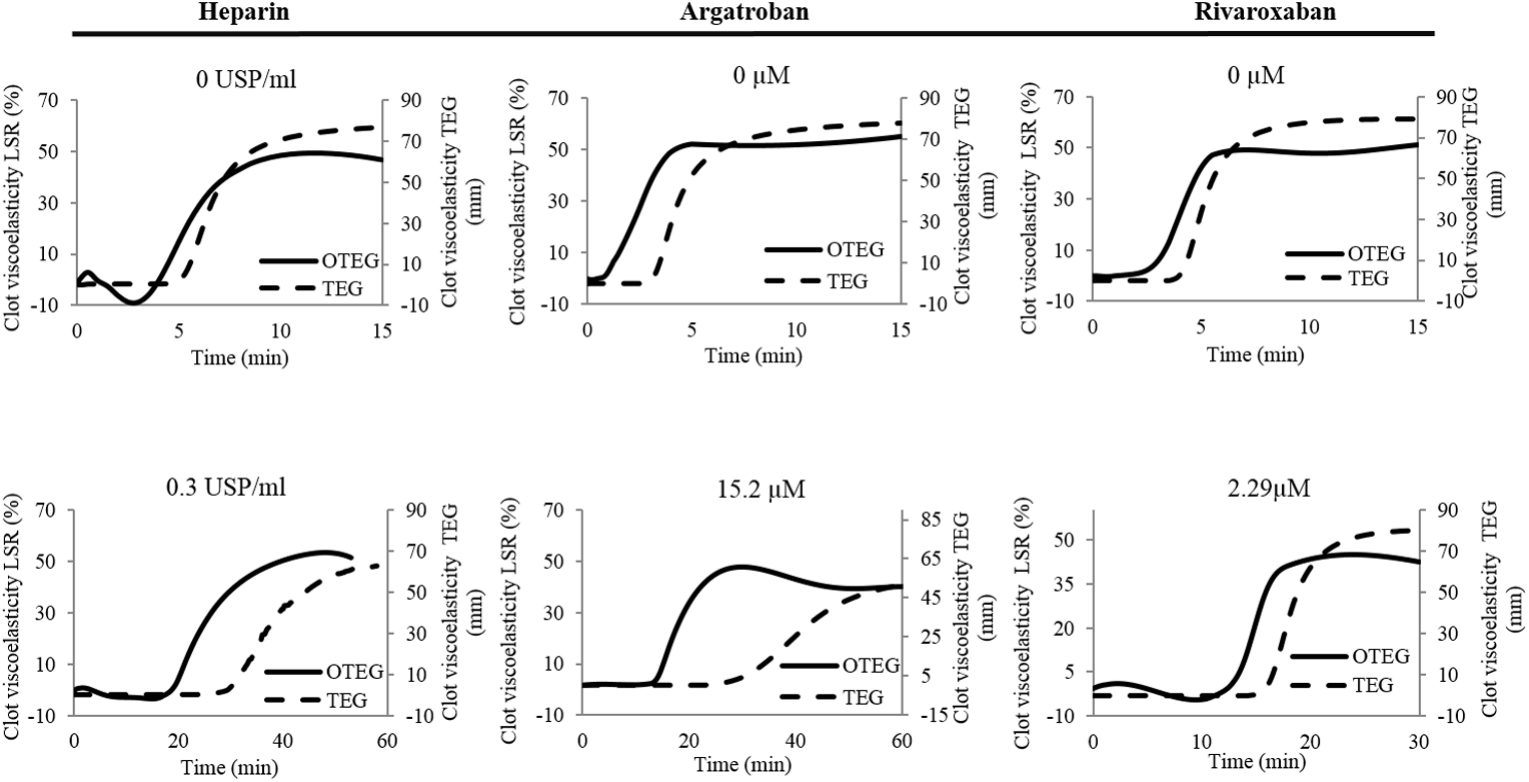
Dose-dependent clot viscoelasticity profiles measured by LSR and TEG. Clot formation of recalcified and kaolin-activated citrated whole blood was measured in the presence of heparin (0.3 USP/ml), argatroban (15.2μM) or rivaroxaban (2.29 μM) and compared with control samples (samples without anticoagulants). In all cases, dose-dependent changes in clot viscoelasticity profiles are noted by both LSR (solid curves) and TEG (dashed curves). The LSR profile trends closely mirror those measured by standard TEG.

**Fig 4:**
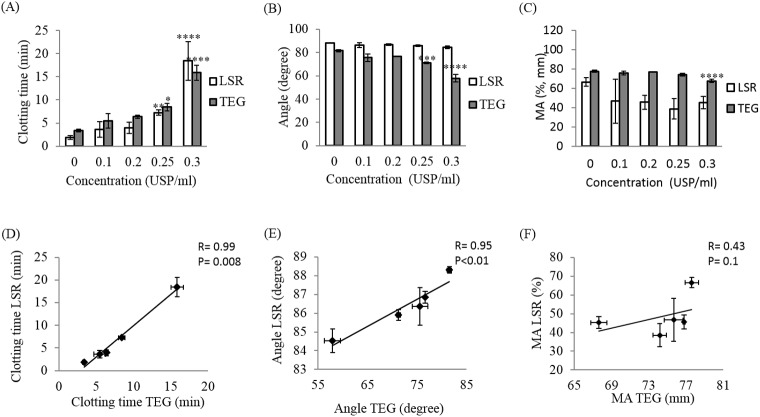
Effect of heparin on LSR and TEG coagulation parameters. Blood coagulation parameters including the clotting time (R+K), the clot progression (angle) and the maximum amplitude (MA) were measured using LSR and TEG for 20–60 minutes following kaolin-activation of swine whole blood samples spiked with heparin at concentration 0 (control), 0.1, 0.2, 0.25, 0.3 USP/ml (A-C). Linear regression analysis between TEG and LSR coagulation parameters at each concentration was performed (D-F). Each data point represents the mean of three replications ± standard deviation (SD) (histograms) or standard error of the mean, SEM (linear regression). Values were compared between control samples (without treatment) and heparin treated samples using ANOVA followed by the Tukey’s method for multiple comparisons post-tests. * p<0.05,** p<0.01, *** p<0.001, **** p<0.0001.

**Fig 5:**
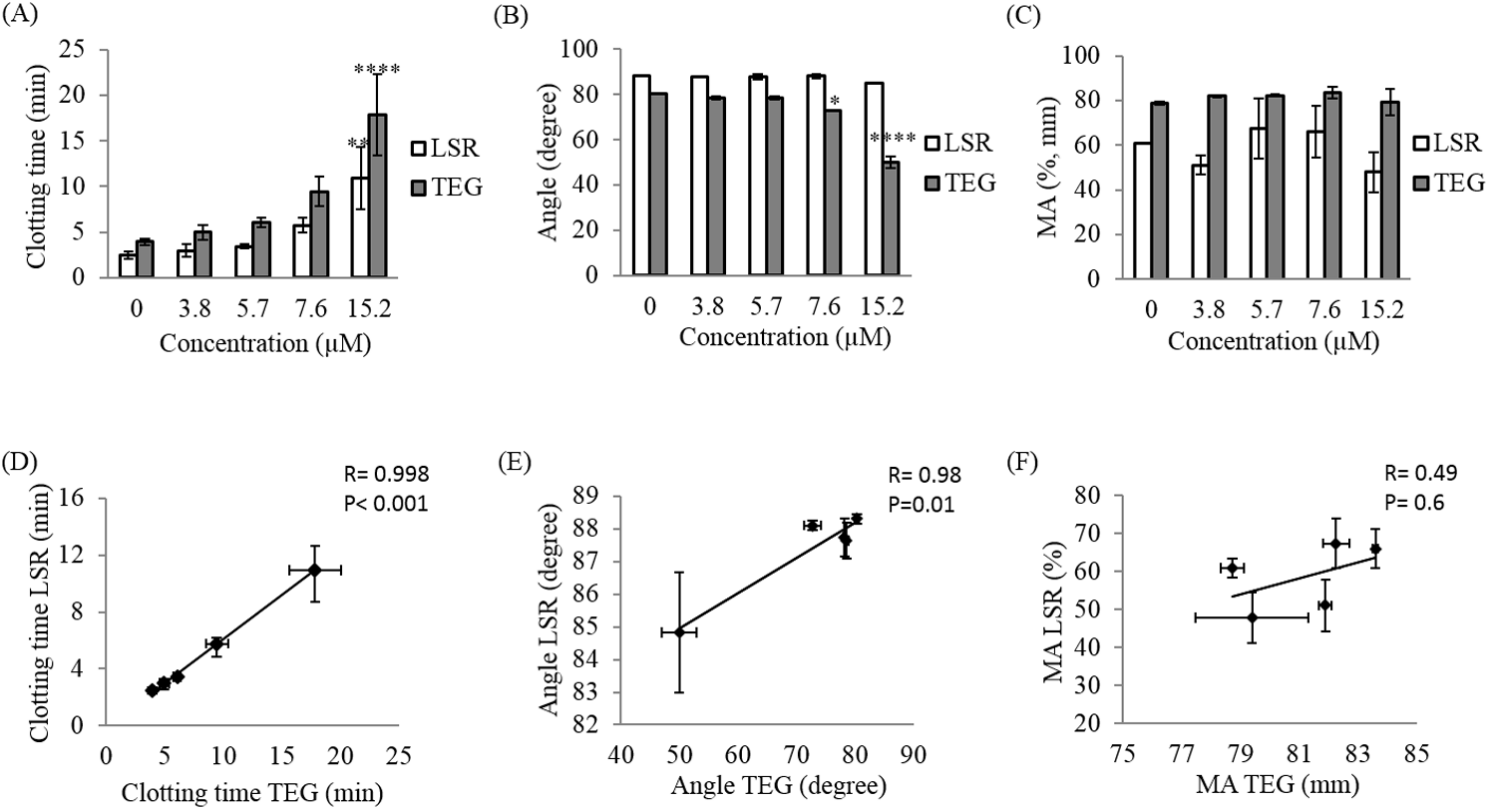
Effect of argatroban on LSR and TEG coagulation parameters. Kaolin-activated swine blood spiked with 0 (control), 3.8, 5.7, 7.6, 15.2 μM argatroban was measured for 20–50 minutes and blood coagulation parameters including the clotting time (R+K), the clot progression (angle) and the maximum amplitude (MA) were extracted for each concentration (A-C). Correlation between LSR and TEG was evaluated using linear regression analysis (D-F). Each data point represents the mean of three replications ± SD (histograms) or standard error of the mean, SEM (linear regression). Values were compared between control samples (without treatment) and argatroban treated samples using ANOVA followed by the Tukey’s method for multiple comparisons post-tests. * p<0.05, ** p<0.01, *** p<0.001, **** p<0.0001.

**Fig 6:**
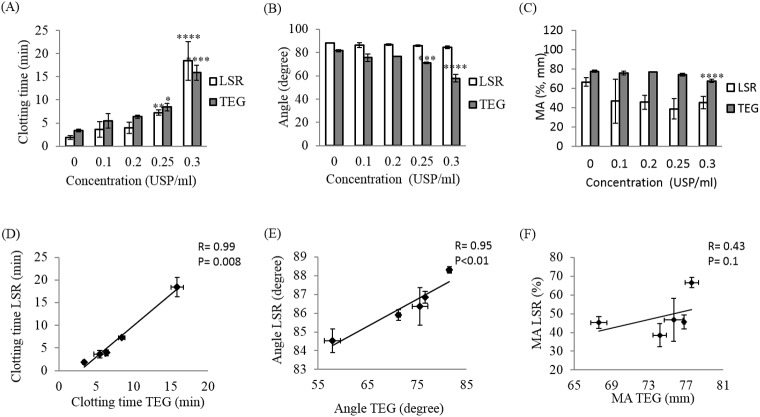
Effect of rivaroxaban on coagulation parameters extracted from LSR and TEG. Kaolin-activated swine blood spiked with 0 (control), 0.46, 1.15, 1.73, 2.29 μM rivaroxaban was measured for 30–45 minutes and blood coagulation parameters including the clotting time (R+K), the clot progression (angle) and the maximum amplitude (MA) were extracted at these concentrations (A-C). Linear regression analysis was performed to analyze correlation between TEG and LSR (B-F). Each data point represents the mean of three replications ± SD (histograms) or standard error of the mean, standard error of the mean, SEM (linear regression). Values were compared between control samples (without treatment) and rivaroxaban treated samples using ANOVA followed by the Tukey’s method for multiple comparisons post-tests. * p<0.05, ** p<0.01, *** p <0.001, **** p<0.0001.

Figs3–6 show the results of studies conducted to evaluate the accuracy and sensitivity of LSR to dose-dependent anticoagulation in swine blood samples. In each case, LSR amplitude curves and coagulation parameters were compared with corresponding coagulation profiles and parameters obtained from TEG (Figs 3–6). Fig 3 shows representative LSR and TEG coagulation profiles in swine blood obtained following treatment with heparin, argatroban and rivaroxaban at various doses. As shown, in all cases coagulation profiles measured using LSR closely matched the corresponding trends measured by TEG. The anticoagulant agents affected the LSR and TEG coagulation profiles in a dose-dependent manner by prolonging the clotting time, in particular. The results obtained for each anticoagulant are detailed below.

#### Heparin

Treatment with heparin at concentrations varying from 0–0.3 USP/ml caused an exponential increase of the clotting time with values ranging from 1.87 ± 0.45 min to 18.41 ± 4.23 min (p<0.0001) for LSR, and from 3.37 ± 0.32 min to 15.88 ± 1.65 min (p<0.0001) for TEG (Figs 3 and 4A). Furthermore, increasing heparin concentration decreased the α-angle in both LSR and TEG profiles from 88.3±0.30° to 84.53 ± 1.24° (p<0.0001) and from 81.53 ± 0.75 to 57.95 ± 3.26 (p<0.0001), respectively (Fig 4B). LSR measurements of clotting times and angles demonstrated excellent correlation with the corresponding TEG measurements with a correlation coefficient r = 0.99, p<0.001 for clotting times (Fig 4D) and r = 0.95, p<0.01 for α-angles (Fig 4E). Changes in MA were observed in LSR and TEG related with heparin dose (Fig 4C). Particularly, with increasing heparin concentration, a trend towards reduction in the MA from 66.56 ± 4.65% at 0 USP/ml to 45.18 ± 6.27% at 0.3 USP/ml was observed using LSR; these differences however were not statistically significant (p = 0.1). In contrast, TEG displayed a significant reduction in MA from 77.63 ± 1.23mm at 0 USP/ml to 67.65 ± 1.78mm at 0.3 USP/ml (p<0.0001), thereby explaining the lower MA correlation of r = 0.43 between both devices (p = 0.5) (Fig 4C and 4F). This discrepancy between LSR and TEG measurements of MA could be due to differences in the properties of the clot formed under quiescent (in LSR) and under high strain conditions (in TEG) as detailed below in the Discussion section.

#### Argatroban

Similar to heparin, treatment with argatroban significantly prolonged clotting times in both LSR and TEG with values ranging from 2.48 ± 0.37 min to 10.9±3.4 min for LSR (p<0.01) and 3.93±0.32 min to 18.85±4.45 min for TEG (p<0.0001) (Figs 3 and 5A). While the α-angle was largely maintained with argatroban concentration, at a concentration above 7.6 μM a slight reduction from 88.31°±0.25° to 84.83°±3.67° (p = 0.2) was observed in LSR and a significant decrease from 80.33±0.70° to 50±5.82° (p<0.0001) was measured by TEG (Fig 5B). Clot MA values were largely preserved in both LSR and TEG measurements with no significant differences observed between doses (p = 0.1) (Fig 5C). A strong correlation was observed between LSR measurements of clotting time (r = 0.99; p<0.001) and α-angles (r = 0.95, p<0.01) versus the corresponding TEG measurements; however, the absence of a dose-dependent modulation in MA using both methods led to a low correlation of r = 0.49 (p = 0.6) for this parameter versus TEG (Fig 5D–5F).

#### Rivaroxaban

Rivaroxaban, an oral direct factor Xa inhibitor, significantly prolonged the clotting time from 3.78 ± 0.68 min to 12.96 ± 1.50 min in LSR and 4.53 ± 0.30 min to 17.53 ± 1.29 min in TEG in a dose-dependent manner (p<0.0001 for both technologies) (Figs 3 and 6A). Similar to heparin and argatroban, LSR and TEG measurements of clotting time showed a strong correlation of r = 0.99 (p<0.01). No dose-dependent differences were detected in the α-angle values measured by LSR (p = 0.3), whereas, an increase in rivaroxaban concentration caused a small, reduction in TEG α-angle from 81.43°±0.67° to 71.8°±1.1° (p = 0.02) (Fig 6B). As a result, the correlation between LSR and TEG measurements of α-angles was not statistically significant (r = 0.77, p = 0.1). As observed in Fig 6C, both LSR and TEG measurements of MA remained unchanged by rivaroxaban concentration (p = 0.5). Thus, in the absence of dose-dependent variation, a poor correlation in LSR and TEG measurements of MA was however observed (r = 0.49, p = 0.4) (Fig 6D–6F).

#### Assessment of haemodilution in swine blood using LSR and TEG

**Fig 7:**
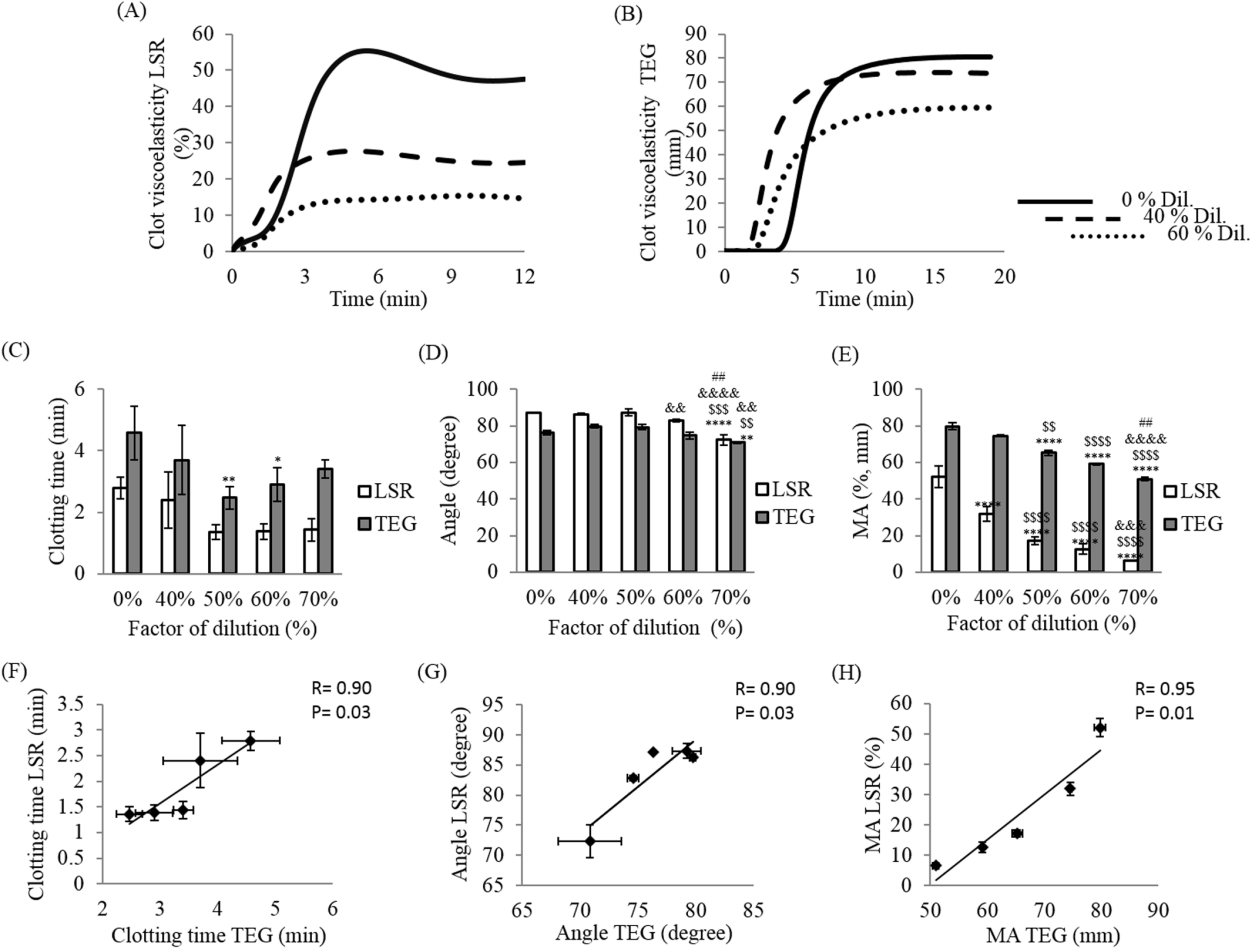
Effect of haemodilution on LSR and TEG clot viscoelasticity profiles and parameters. Recalcified and kaolin-activated swine whole blood diluted at 0 (undiluted blood sample) 40, 50, 60 or 70% were measured using LSR (A) and TEG instruments (B) and blood coagulation parameters such as the clotting time (R+K) (C), the clot progression (angle) (D) and the maximum amplitude (MA) (E) were extracted at various haemodilution concentrations. Linear regression analysis comparing blood coagulation parameters R+K (F) angle (G), and MA (H) between LSR and TEG are presented. Each data point represents the mean of three replications ± SD (histograms) or standard error of the mean, SEM (linear regression). Comparisons were done using ANOVA followed by the Tukey’s method for multiple comparisons. (*, 0%-…); ($, 40%-…); (&, 50%-…); (#, 60–70%). * p<0.05, ** p<0.01, *** p<0.001, **** p<0.0001.

Fluid resuscitation, a primary approach of managing haemorrhage caused by excessive anticoagulation in patients, can lead to further coagulation impairment via dilution of clotting factors. Therefore, we assessed the capability of LSR for detecting coagulation impairments caused by serial haemodilution via comparison with TEG. As observed in Fig 7A, haemodilution significantly affected LSR coagulation profiles and parameters in a dose-dependent manner similar to TEG (Fig 7B). For dilutions greater than 50%, a slight increase in clotting time was measured: from 1.38±0.25 min to 1.43±0.36 min for LSR and from 2.9±0.56 to 3.4±0.30 minutes for TEG (p = 0.09) (Fig 7C). However, both LSR and TEG measured steady decreases in clotting times (R+K) for serial haemodilution of up to 50%; from 2.79±0.36 min to 1.35±0.25 min for LSR (p = 0.08) and from 4.57±0.86 min to 2.47±0.38 min for TEG (p = 0.04). Furthermore, clotting time (R+K) measured by LSR for the diluted blood samples demonstrated strong correlation with the corresponding TEG measurements (r = 0.90; p = 0.03) (Fig 7F). Contrary to clotting time results, below 50% haemodilution, both LSR and TEG presented no significant change in the α-angle. For haemodilution levels beyond 50% however, a slight decrease in α-angle was noted from 82.83±0.84° to 72.33±2.74° (p<0.01) by LSR and from 74.60±2.12° to 70.87±0.46° (p<0.01) by TEG (Fig 7D). LSR measurements of α-angle demonstrated a strong correlation (r = 0.90; p = 0.03) versus TEG (Fig 7G). Interestingly, the clot stiffness parameter, MA, was most susceptible to haemodilution in a dose-dependent manner. While both devices presented a diminution in MA with increasing haemodilution, LSR detected a larger MA modulation in comparison to TEG (Fig 7A and 7B). A steady decrease in MA from 52.09±5.92 to 6.54±0.22 (p<0.0001) was noted by LSR and MA varied from 79.77±1.75 to 50.97±0.92 (p<0.0001) in TEG. This resulted in an excellent correlation measured between LSR and TEG values of MA (r = 0.95, p = 0.01) (Fig 7E and 7H).

## Discussion

The complexity of anticoagulant therapy affected by a narrow therapeutic window is responsible for a high number of adverse reactions such as haemorrhage and thrombotic events [1,32]. Frequent monitoring of coagulation status is therefore crucial to ensure appropriate anticoagulant dosing and to maintain a critical balance between coagulation and bleeding [33]. Point-of-care coagulation testing may offer a simplified, convenient, and inexpensive alternative to traditional laboratory monitoring with rapid, comprehensive, and real-time reporting of coagulation results [33,34]. Here, we have demonstrated for the first time, the capability of LSR, a novel optical coagulation sensing technology, for assessing the treatment-response of common anticoagulants such as warfarin, heparin, argatroban, and rivaroxaban in anticoagulated patients and in swine. In addition, the influence of haemodilution on the LSR coagulation profile was also evaluated to test the feasibility of potentially detecting coagulation impairments caused by fluid resuscitation.

To assess the measurement accuracy for anticoagulation sensing, LSR parameters were compared to those measured with its mechanical counterpart, TEG, a mechanical sensor shown to be useful in evaluating the coagulation status of patients treated with heparin or direct thrombin and FX inhibitors [11,22,35]. In the clinical testing studies reported in Fig 2, LSR metrics were also compared to CCT results of aPTT, PT and INR that have been used as the clinical standard of care to assess patients on warfarin therapy.

The objective of this study was to validate the capability of the LSR technology to assess the four broad classes of anticoagulant drugs most frequently used in patients. In this study, we chose one drug from each of the four broad classes of anticoagulants to demonstrate the capability of the technology in monitoring dose-dependent anticoagulation for each mechanism of action. In our future work, additional studies will be performed to include additional anticoagulant drugs from similar classes, such as dabigatran (direct thrombin inhibitor), apixaban (Factor X inhibitor) and Lovenox (low molecular weight heparin). Given that the results of the current study establish the capability for monitoring all of the four major anticoagulant classes, we expect similar results for other anticoagulant drugs from these classes in future studies.

Warfarin and other coumarin derivatives exert anticoagulant effects by limiting hepatic production of functional vitamin K-dependent coagulation factors including prothrombin, FIX, FX and FVII, the latter being the first protein to be depleted [36]. Warfarin treatment commonly assessed using aPTT and INR (normalised ratio of PT), requires frequent monitoring to determine the proper anticoagulant effect. In this study, LSR clotting time presented a strong correlation with aPTT and good correlation to PT and INR (Fig 2D and 2E). The relatively moderate correlation of LSR clotting time with PT/INR versus aPTT might be explained by differences in the modes of coagulation activation utilised by the different assays. Similar to LSR and TEG, the aPTT assay uses kaolin or celite to activate clot formation via the intrinsic and common pathway, whereas PT/INR employs tissue factor utilizing extrinsic and common pathway catalysers for coagulation [36,37]. In other words, LSR, aPTT and TEG assays are more sensitive to coagulation cascades resulting from kaolin activation via the intrinsic and common pathways, explaining the high correlation between LSR clotting time and aPTT compared to PT/INR (Fig 2C and 2F) [38]. Although kaolin activation was employed in this study to evaluate warfarin therapy using LSR, the use of tissue factor as a clotting activator could just as easily be employed in conjunction with LSR to allow for a more accurate evaluation of PT/INR values.

We further observed that the anticoagulation effects of heparin, argatroban and rivaroxaban varied markedly in a concentration-dependent manner (Figs 3–6) and in concordance with prior studies using other devices [19,39]. In LSR, the elongation of clotting time was closely associated with anticoagulant dose similar to TEG. Clotting time variations for heparin and argatroban presented similar trends, with small increases at low doses followed by an exponential increase in clotting time at higher concentrations (Figs 4A and 5A). By enhancing antithrombin activity heparin indirectly and irreversibly catalyses inactivation of FXa, thrombin, and other coagulation factors, thus cumulatively modulating the intrinsic, extrinsic and common pathways of coagulation [40,41]. These factors may explain the larger effect of heparin on the clotting time in comparison to argatroban and rivaroxaban at high concentration (Figs 4A–6A). Nevertheless, heparin also binds to plasma proteins, macrophages and platelet factor 4 (PF4) in a non-specific manner, reducing their availability and prompting a low detection sensitivity in clotting time by both LSR and TEG at lower heparin concentration [40,42,43]. Conversely, argatroban and rivaroxaban are direct inhibitors, and mainly regulate the common pathway of the coagulation cascade by specifically inhibiting thrombin and FX respectively [7,44,45]. In particular, with a dissociation rate constant (K_off_) 1000-fold slower and an inhibition constant 100-fold weaker than argatroban, rivaroxaban is a more potent anticoagulant likely explaining the longer clotting time even for low molar concentrations (Figs 4A–6A) [7,44,46].

Despite the importance of clotting time in assessing the therapeutic effect of anticoagulant drugs, clinical evidence indicates that anticoagulation management may be further improved via the comprehensive assessment of downstream processes such as clot progression and fibrin polymerization characterized by the α-angle and MA respectively which cannot be easily assessed by CCT [11,20,39]. For instance, studies have shown that increased clot stiffness may yet be elevated in some patients undergoing vitamin K antagonist therapy raising the risk of thrombosis. While, on the flip side, compromised clot strength and increased clot breakdown (hyperfibrinolysis) may be associated with long-term anticoagulant use thus elevating the risk of hemorrhage [21,47]. Since LSR provides the capability to quantitatively assess fibrin clot progression (via α-angle), stabilisation (via MA) and clot lysis occurring after the initial clotting time is reported, this new technology may likely improve clinical management of anticoagulation therapy in patients.

Our results obtained from spiked swine blood showed that the anticoagulant type and dose modulated the LSR α-angle, whereas MA measured using LSR remained largely unchanged (Figs 4–6). Since the MA parameter is largely influenced by thrombin modulation, the normal MA observed with LSR in the presence of all studied anticoagulants is consistent with the limitation in their mode of action. Other studies have reported that the anticoagulation effect of heparin may be reduced by its inability to inhibit thrombin already bound to the clot [48–50]. Similarly, argatroban only antagonizes pre-formed thrombin and is unable to regulate new formation of thrombin [32,50]. Moreover, argatroban’s antithrombotic effect involves a reversible attachment to thrombin active sites therefore allowing thrombin function recovery, which could create a loophole in which some thrombotic activity is maintained likely causing MA values to remain stable even with increased treatment dose consistent with our results [32,50,51]. Similarly, rivaroxaban may enable the recovery of FX activity over time allowing for normal clot formation and therefore eliciting an MA clot stiffness value that is normal or largely unchanged [19,50]. Other studies have similarly reported that stable clots in whole blood can be generated in presence of less than 4% activated thrombin [52,53]. In contrast to these prior studies however, TEG reports significant changes in α-angle by all anticoagulants and MA by heparin, which is likely due to contact-based coagulation sensing mechanism in TEG. In other words, TEG modifies the clot structure by physically manipulating the clot during measurement thereby likely leading to a substantially weaker clot and subsequently lower α-angle and MA [54–56]. Since LSR on the other hand is a non-contact method that measures clot viscoelasticity without physical manipulation of the clot, this new optical approach may likely more accurately recapitulate the complex *in vivo* environment of whole blood coagulation.

In addition to anticoagulation testing, point of care coagulation testing using LSR may be relevant in several clinical settings to guide fluid resuscitation treatment in order to maintain patient normovolaemia in situations of severe haemorrhage caused by anticoagulant overdose [54]. Excessive haemodilution has been linked to impairment in coagulation factors [6,54,57]; therefore, LSR testing to detect and monitor coagulation abnormalities caused by haemodilution may be invaluable in managing bleeding patients with anticoagulant overdose. In this study, citrated swine blood was serially diluted with 0.9% NaCl isotonic to mimic the transfusion of crystalloids solution and evaluate the effects of haemodilution using LSR compared with TEG. LSR parameters were significantly affected by haemodilution and were closely related with the corresponding TEG results in all cases. In particular, the LSR MA was significantly reduced by dilution similar to TEG, suggesting the presence of weak fibrin clots with excessive haemodilution. The clotting time, however, presented an initial decrease followed by a rapid increase at higher levels of dilution with both LSR and TEG. The trend observed in the clotting time could be due to the modulation of both pro-coagulant and anticoagulant factors by excessive haemodilution [6,57,58]. The reduction of thrombin activity due to haemodilution can be partially compensated for by a reduction in the activity of antithrombin and other pro-coagulant inhibitors. Antithrombin, one of the main anticoagulants *in vivo*, has a higher sensitivity to haemodilution in comparison to thrombin and other pro-coagulants [6,57]. Consequently, it has been suggested in other studies that the reduction of antithrombin activity prolongs the half-life of thrombin and activated-FX, which may contribute to shortening of clotting time and acceleration of clot progression at 50% dilution consistent with our observations in this study [6]. Furthermore, during the course of haemorrhage and massive resuscitation treatment, fibrinogen is the first coagulation factor to reach critically low levels (<100 mg/dl) [57,59]. Since clot stabilization is highly dependent on fibrinogen levels in blood [14,60], dilution of fibrinogen levels significantly diminishes MA at all haemodilution levels. We observed a strong correlation in LSR and TEG coagulation parameters in assessing anticoagulation or dilution treatments. The error bars observed in the measurements for both technologies are likely due to the inherent heterogeneity of the blood sample which can change over the duration of the experiment. Furthermore, the relatively small number of repetitions (N = 3) in each group may also contribute to the observed variability. By increasing the number of repetitions measured for each anticoagulant type and dose to account for blood heterogeneity in the future we will likely lower the standard deviations observed in both approaches. Furthermore, LSR appears to be more sensitive to changes in clot MA during haemodilution (Fig 7E), and could detect small changes in MA created even by low levels of dilution.

While the strength of the fibrin network mainly depends on factor XIII and fibrinogen, platelets participate in the overall clot strength by binding with the fibrin network. Several studies, have shown that both high platelets count or fibrinogen concentrations influence the MA, with changes in both components modulating blood clot stiffness [61,62]. In other words, LSR measures clot viscoelasticity by quantifying minute, nanometer-scale Brownian displacements (on the order of the light wavelength) of light scattering particles in clotting blood and is therefore exquisitely sensitive to small changes in clot viscoelasticity and platelets aggregation. These factors may explain the higher measurement sensitivity to haemodilution observed by LSR in contrast with TEG [15]. Thus, the ability of LSR to measure the viscoelastic modulus and the changes in particles size during blood clot formation might provide a more accurate and physiologically relevant view of the coagulation process than conventional coagulation tests alone.

In the first part of the current study, the clinical testing results presented above established the capability and utility of the LSR technology for future clinical use in patients. In the second part of the study to evaluate anticoagulant dose-dependence, whole blood from swine was used to evaluate LSR sensitivity. The use of fresh swine whole blood rather than human whole blood was primarily motivated by the requirement for substantially large blood volumes needed for testing the dose-dependency of multiple anticoagulants and hemodilution via triplicate measurements using both LSR and TEG. Since collection of large volumes of patient blood samples for dose-dependent anticoagulation testing was impractical, swine blood was spiked and tested with LSR and TEG. However, the dose-dependant response to anticoagulation and haemodilution in swine whole blood may differ from human whole blood under similar conditions, and therefore the direct extrapolation of these results to human subjects may be slightly limited. It is important to note at the same time this limitation was addressed in this paper by the comparison of LSR results obtained from patients on warfarin therapy with TEG and with laboratory-based CCTs, which presented a good correlation of coagulation parameters in these cases. In future work, blood samples will be conducted from normal human volunteers to measure LSR sensitivity to anticoagulant and hemodilution dose.

In order to assess the individual contribution of fibrin polymerization and platelet aggregation to the clot strength, it is possible to utilize a fibrinogen functional assay that diminishes the effects of platelet aggregation on the measured clot strength by inhibiting the conformational changes of platelets glycoprotein IIb/IIIa receptors [61,62]. Additionally, the effects of platelet aggregation can be solely studies by conducting OTEG in citrated blood and activating platelets using an agonist such as ADP. These assays could be readily used with the LSR technology to assess the influence of the fibrin network or platelet aggregation on the MA in isolation [61,62].

## Conclusion

LSR provides rapid assessment of anticoagulation status in whole blood in a non-contact manner enabling measurements in near-physiological conditions. Furthermore, the pilot clinical testing studies reported in this paper demonstrate the accuracy and utility for anticoagulation sensing in patients using small blood volumes within minutes in real-time. Thus far, the clinical PoC adoption of viscoelastic assay approaches such as TEG has been limited due to their large size, high cost and complexity of use [11]. In contrast, LSR utilises an inexpensive diode laser and CMOS camera with no moving mechanical parts offering the opportunity for fabricating a low-cost, palm-sized tool for anticoagulation monitoring at the patient’s bedside, in the physician’s office or for patient self-testing. LSR measurements were performed in this study using inexpensive off-the-shelf disposable cartridges that utilized 127 μl of whole blood. We are currently developing methods to reduce the LSR blood volume to 40 μl or less, which will open the powerful opportunity for self-testing of multiple coagulation parameters via a finger-stick blood draw in the patient’s home.

## Supporting information

S1 Table Effect of heparin on LSR and TEG coagulation parameters. Each data point represents the mean of three replications ± standard deviation (SD).
(DOCX)

S2 Table Effect of argatroban on LSR and TEG coagulation parameters. Each data point represents the mean of three replications ± standard deviation (SD).
(DOCX)

S3 Table Effect of rivaroxaban on LSR and TEG coagulation parameters. Each data point represents the mean of three replications ± standard deviation (SD).
(DOCX)

S4 Table Effect of dilution on LSR and TEG coagulation parameters. Each data point represents the mean of three replications ± standard deviation (SD).
(DOCX)

## Abbreviations

aPTT: Activated partial thromboplastin time
ACT: Activated clotting time
CaCl_2_: Calcium Chloride
GSER: Generalised Stokes Einstein Relation
INR: International normalised ratio
K: Kinetic time
LSR: Laser Speckle Rheology
MA: Maximum amplitude
MSD: Mean square displacement
NaCl: Sodium Chloride
PF4: Platelet factor- 4
POC: Point of Care
PT: Prothrombin time
R: Reaction time
ROI: Region of interest
ROTEM: Rotational thromboelastometry
TEG: Thromboelastography
