# Supplementary material for: Optical sensing of anticoagulation status: Towards point-of-care coagulation testing
Source: PLoS One. 2017 Aug 3;12(8):e0182491. doi: 10.1371/journal.pone.0182491 (PMC5542647; doi:10.1371/journal.pone.0182491)
Supplement: S1 Table — Each data point represents the mean of three replications ± standard deviation (SD). (DOCX) [file pone.0182491.s001.docx]

**S1 Table 1:** **Effect of heparin on LSR and TEG coagulation parameters**

| Heparin  concentration  (USP/ml) | LSR  Clotting time  (Min) | TEG  Clotting time  (Min) | LSR  Angle  (degree) | TEG  Angle  (degree) | LSR  MA  (%) | TEG  MA  (mm) |
| --- | --- | --- | --- | --- | --- | --- |
| 0 | 1.87±0.45 | 3.37±0.32 | 88.30±0.30 | 81.53±0.75 | 66.56±4.65 | 77.63±1.23 |
| 0.1 | 3.63±1.68 | 5.47±1.54 | 86.36±2.00 | 75.57±3.14 | 46.61±22.92 | 75.70±2.00 |
| 0.2 | 3.93±1.20 | 6.40±0.35 | 86.85±0.62 | 76.63±0.06 | 45.52±7.31 | 76.80±0.20 |
| 0.25 | 7.26±0.61 | 8.45±0.78 | 85.90±0.51 | 71.25±0.64 | 38.54±10.55 | 74.20±1.27 |
| 0.3 | 18.41±4.23 | 15.87±1.65 | 84.53±1.24 | 57.95±3.26 | 45.18±6.27 | 67.65±1.78 |

Each data point represents the mean of three replications ± standard deviation (SD).
