# Supplementary material for: Optical sensing of anticoagulation status: Towards point-of-care coagulation testing
Source: PLoS One. 2017 Aug 3;12(8):e0182491. doi: 10.1371/journal.pone.0182491 (PMC5542647; doi:10.1371/journal.pone.0182491)
Supplement: S2 Table — Each data point represents the mean of three replications ± standard deviation (SD). (DOCX) [file pone.0182491.s002.docx]

**S2 Table 2:** **Effect of argatroban on LSR and TEG coagulation parameters**

| Argatroban  concentration  (µM) | LSR  Clotting time  (Min) | TEG  Clotting time  (Min) | LSR  Angle  (degree) | TEG  Angle  (degree) | LSR  MA  (%) | TEG  MA  (mm) |
| --- | --- | --- | --- | --- | --- | --- |
| 0 | 2.48±0.37 | 3.13±0.32 | 88.31±0.25 | 80.33±0.70 | 60.99±4.26 | 78.73±0.68 |
| 3.8 | 2.97±0.65 | 4.1±0.83 | 87.64±1.06 | 78.60±0.65 | 51.11±13.45 | 81.90±0.42 |
| 5.7 | 3.43±0.24 | 5.07±0.47 | 87.75±1.01 | 78.27±0.35 | 67.46±11.43 | 82.27±0.76 |
| 7.6 | 5.75±0.77 | 7.90±1.30 | 88.10±0.26 | 72.80±2.55 | 66.045±8.99 | 83.60±0.14 |
| 15.2 | 10.92±3.40 | 13.17±3.80 | 84.83±3.67 | 50.00±5.82 | 47.87±11.50 | 79.40±3.92 |

Each data point represents the mean of three replications ± standard deviation (SD).
