# Supplementary material for: Optical sensing of anticoagulation status: Towards point-of-care coagulation testing
Source: PLoS One. 2017 Aug 3;12(8):e0182491. doi: 10.1371/journal.pone.0182491 (PMC5542647; doi:10.1371/journal.pone.0182491)
Supplement: S3 Table — Each data point represents the mean of three replications ± standard deviation (SD). (DOCX) [file pone.0182491.s003.docx]

**S3 Table 3:** **Effect of rivaroxaban on LSR and TEG coagulation parameters**

| Rivaroxaban  concentration  (µM) |  | LSR  Clotting time  (Min) | TEG  Clotting time  (Min) | LSR  Angle  (degree) | TEG  Angle  (degree) | LSR  MA  (%) | TEG  MA  (mm) |
| --- | --- | --- | --- | --- | --- | --- | --- |
| 0 |  | 3.78±0.82 | 4.53±0.31 | 87.49±0.24 | 81.43±0.67 | 53.52±3.05 | 80.09±1.42 |
| 0.46 |  | 6.80±1.47 | 6.87±0.21 | 88±0.65 | 79.03±0.91 | 54.16±11.63 | 78.06±3.10 |
| 1.15 |  | 7.34±0.89 | 8.43±1.01 | 87.46±1.18 | 76.65±1.02 | 43.93±14.12 | 76.75±2.11 |
| 1.73 |  | 9.98±0.86 | 13.53±2.20 | 87.23±0.87 | 74.7±0.42 | 59.80±6.07 | 82.83±3.26 |
| 2.29 |  | 12.96±1.50 | 17.53±1.29 | 86.90±0.60 | 71.8±1.10 | 48.27±4.30 | 83.00±2.55 |

Each data point represents the mean of three replications ± standard deviation (SD).
