# Supplementary material for: Optical sensing of anticoagulation status: Towards point-of-care coagulation testing
Source: PLoS One. 2017 Aug 3;12(8):e0182491. doi: 10.1371/journal.pone.0182491 (PMC5542647; doi:10.1371/journal.pone.0182491)
Supplement: S4 Table — Each data point represents the mean of three replications ± standard deviation (SD). (DOCX) [file pone.0182491.s004.docx]

**S4 Table 4:** **Effect of dilution on LSR and TEG coagulation parameters**

| Factor of dilution | LSR  Clotting time  (Min) | TEG  Clotting time  (Min) | LSR  Angle  (degree) | TEG  Angle  (degree) | LSR  MA  (%) | TEG  MA  (mm) |
| --- | --- | --- | --- | --- | --- | --- |
| 0 | 2.79±0.36 | 4.57±0.87 | 87.17±0.09 | 76.35±1.07 | 52.09±5.92 | 79.77±1.75 |
| 40% | 2.40±0.92 | 3.70±1.11 | 86.28±0.46 | 79.77±1.12 | 31.92±3.92 | 74.57±0.51 |
| 50% | 1.36±0.25 | 2.47±0.37 | 87.08±2.56 | 79.23±1.55 | 17.21±17.21 | 65.23±1.50 |
| 60% | 1.38±0.25 | 2.90±0.56 | 82.83±0.84 | 74.60±2.12 | 12.66±12.66 | 59.13±0.47 |
| 2.29% | 1.52±0.36 | 3.40±0.29 | 74.83±4.74 | 70.87±0.46 | 6.92±6.92 | 50.97±0.92 |

Each data point represents the mean of three replications ± standard deviation (SD).
